# Non-SHEF2 Model related containment and control measures against COVID-19 in Africa

**DOI:** 10.11604/pamj.supp.2020.35.24061

**Published:** 2020-06-21

**Authors:** Frankline Sevidzem Wirsiy, Claude Ngwayu Nkfusai, Fala Bede, Rosette Boseme Nzoyom, Samuel Nambile Cumber

**Affiliations:** 1 Department of Public Health and Hygiene, Faculty of Health Sciences, University of Buea, Buea, Cameroon; 2 Saint Louis University Institute of Health and Biomedical Sciences, Douala, Cameroon; 3 Georgetown University´s Center for Global Health Practice and Impact (CGHPI), TIDE-Cameroon Program, Yaounde, Cameroon; 4 Department of Public Health, School of Nursing and Public Health, University of Kwa-Zulu Natal, Durban, South Africa; 5 Georgetown University´s Center for Global Health Practice and Impact (CGHPI), TIDE-Cameroon Program, Bertoua, Cameroon; 6 Department of Nursing, Grand Canyon University, Arizona United State of America; 7 Bridgeport Hospital Capitole Hill, Washington DC, United State of America; 8 Postdoctoral Fellow, Centre for Health Systems Research & Development, University of the Free State, Bloemfontein, South Africa; 9 Office of the Dean, Faculty of Health Sciences, University of the Free State, Bloemfontein, South Africa; 10 School of Health Systems and Public Health, Faculty of Health Sciences, University of Pretoria, Pretoria, South Africa

**Keywords:** Non-SHEF2 model, TITHQC2, COVID-19, containment, control measures, Africa

## Abstract

COVID-19 Pandemic has the potential to overwhelm the underserved health care systems of African countries characterized by inadequate infrastructure and too few medical personnel. In responding to the COVID-19 Pandemic, many African countries are using a combination of containment and mitigation activities but in this commentary, we focus on what we term the Non-SHEF2 (S: Social distancing, H: Hands, E: Elbows, F: Face, F: Feel) model related control and containment measures which include seven key measures against COVID-19 doped ‘TITHQC2’ namely, T: Travel-related measures, I: Information and guidance, T: Treatment; H: Hospital containment measures; Q: Quarantine, C: Community containment measures, C: Case detection and contact tracing. COVID-19 is a reality and demands rapid and decisive action to be taken.

## Commentary

Reducing the incidence, prevalence, morbidity or mortality of Coronavirus disease (COVID-19) to a locally acceptable level in Africa will require a holistic approach and as such warrants additional measures such as the Non-SHEF2 (“SHEF2”- S: Social distancing, H: Hands, E: Elbows, F: Face, F: Feel) model related control and containment measures which include seven unique measures against COVID-19 doped ‘TITHQC2’ namely, T: Travel-related measures, I: Information and guidance, T: Treatment; H: Hospital containment measures; Q: Quarantine, C: Community containment measures, C: Case detection and contact tracing. COVID-19 has the potential to overwhelm the underserved health care systems of African countries characterized by inadequate infrastructure and too few medical personnel. COVID-19 caused by a novel virus called Severe Acute Respiratory Syndrome Coronavirus 2 (SARS-CoV-2) [1] will keep disrupting social life and public health systems, with broad-ranging consequences, if we don’t establish context related measures. SARS-CoV-2, like other emerging high-threat pathogens, have potential of infecting health-care workers in Africa and several other countries around the world [2]. To date, however, in most African countries now, where infection prevention and control was taken seriously, nosocomial transmission has not been a major amplifier of transmission in this Pandemic

In responding to the COVID-19 Pandemic, many African countries are using a combination of containment and mitigation activities with the intention of delaying major surges of patients and levelling the demand for hospital beds, while protecting the most vulnerable from infection, including elderly people and those with comorbidities [1]. Most national response strategies equally include varying levels of contact tracing and self-isolation or quarantine; promotion of public health measures, including handwashing, respiratory etiquette, and social distancing; preparation of health systems for a surge of severely ill patients who require isolation, oxygen, and mechanical ventilation; strengthening health facility infection prevention and control, with special attention to nursing home facilities; and postponement or cancellation of large-scale public gatherings. This paper aims at discussing Non-SHEF2 (“SHEF2”- S: Social distancing, H: Hands, E: Elbows, F: Face, F: Feel) model related control and containment measures which include seven key measures against COVID-19 in Africa doped ‘TITHQC2’ namely, T: Travel-related measures, I: Information and guidance, T: Treatment; H: Hospital containment measures; Q: Quarantine, C: Community containment measures, C: Case detection and contact tracing. Highlighting the distinctive ‘TITHQC2’ features from an African approach of controlling COVID-19 could enhance better public health emergency preparedness and stronger response.

Non-SHEF2 model; the case of (“TITHQC2” containment and control measures against COVID-19 in Africa: a summary of the key features of the Non-SHEF2 model ((“TITHQC2”) to contain and control COVID-19 in Africa is shown in Table 1.

**Table 1:** Non-SHEF2 model (“TITHQC2”) against the COVID-19 pandemic in Africa

Non-SHEF2 Model (“TITHQC2”)	Keys features
**T: Travel-related measures**	• World Health Organization recommends countries experiencing local COVID-19 transmission to screen departing passengers, which includes asking them questions and checking their temperature
	• Travel related measures by the affected countries themselves and also in response to WHO recommendations to detect COVID-19 cases among domestic and international travelers is an important measure to prevent them from travelling within or leaving the country as well as isolate them immediately on entry
**I: Information and guidance on COVID-19**	◾ Africans are more liable to misinformation via social media. This notwithstanding, there is need to communicate the epidemiology and risks of COVID-19 clearly, both to health-care workers and to the general population
	◾ Various means of communication including telephone hotlines, electronic and print media, the internet, exhibitions, advertisements, etc., are used in creating awareness on the fight against COVID-19
	◾ With the rise of COVD-19 pandemic in Africa, huge propaganda machinery is essential to be activated to inform the public. Ways used to motivate and inform the community in Africa to protect themselves and fight against COVID-19 includes but not limited to health educational programs, songs, banners, COVID-19 advertisements on buses and daily press conferences by the governments
**T: Treatment of COVID-19**	◈ There is no specific antiviral treatment recommended for COVID-19, and no vaccine is currently available.
	◈ The treatment is symptomatic, and oxygen therapy represents the major treatment intervention for patients with severe infection.
	◈ African traditional herbal medicines and plants which have been used for centuries across different cultures on the continent and the COVID-19 pandemic in Africa has also triggered a rush for herbal formulas, onions, lemons, and ginger in the belief that they can protect against the virus
**H: Hospital containment measures**	♠ African countries isolate probable and suspect COVID-19 cases, either in a designated health facility or in separate triage facilities
	♠ Many African countries faced acute shortage of medical masks and other protective equipment. Reinstituting infection control, contact tracing and active surveillance in hospitals can contribute significantly in controlling the COVID-19 outbreak
**Q: Quarantine**	♣ Mandatory quarantine of close contacts is a good measure for African countries experiencing local COVID-19 transmission
	♣ The use of isolation is the best way to contain the COVID-19 Pandemic for individual countries
**C: Community containment measures against COVID-19**	◦ Community containment measures aimed at limiting social interaction and movement of people is important in containing COVID-19
	◦ People are asked to wear masks in closed public places.
	◦ There is need to minimize economic impact and social disruption through multisector, international, community- collaborative, bottom-top and narrative inquiry approaches from a socio-ecological model in regards to community containment measures
**C: Case detection and contact tracing of COVID-19**	♦ Early case detection followed by rapid and effective isolation is a key measure to control COVID-19 spread
	♦ Contact tracing helps to limit COVID-19 transmission when the first cases are identified within a country but can be very resource intensive for Africa
	♦ During contact tracing, the contacts of the infected person are generally called up, asked if they're feeling sick, and advised to self-quarantine for a period of time

**T: Travel-related measures:** it is worthy to note that, if a large variety of travel-related restrictive measures were introduced earlier on in Africa, the COVID-19 Pandemic would not have been at its worst. Travel related measures by the affected countries themselves and also in response to WHO recommendations to detect COVID-19 cases among domestic and international travelers is an important measure to prevent them from travelling within or leaving the country as well as isolate them immediately on entry [3]. Fear of getting infected also led to a substantial reduction in travel volume. WHO recommends countries experiencing local COVID-19 transmission to screen departing passengers, which includes asking them questions and checking their temperature [3]. Millions of travelers entering or leaving affected areas via different routes are subjected to thermal scanning using infrared scanners [3]. Earlier attention of African COVID-19 surveillance was at countries’ points of entry, and testing targeted people with a recent travel history to outbreak areas abroad [1]. However, we assert that screening passengers for fever is ineffective, because it doesn’t catch people still in their incubation phase-up to 14 days for COVID-19. It also won’t detect cases that occur in African communities. Patients who came from Asia, Europe and America will likely have interacted with Africans prior to their diagnosis, including household nannies and helps, who often take crowded minibuses to their homes in low-income areas- all perfect conditions for COVID-19 community transmission.

**I: Information and guidance on COVID-19:** Africans are more liable to misinformation via social media. This notwithstanding, there is need to communicate the epidemiology and risks of COVID-19 clearly, both to health-care workers and to the general population among African member states, and to implement infection prevention and control measures that are based on sound scientific principles [4]. Affected African countries hold official press conferences and issued press releases to inform their public and different stakeholders about COVID-19, the status of the outbreak within and outside their countries, about the risk factors and preventive measures and about government actions to counter the Pandemic. Due to some factors ranging from psychological to political, some African countries like Cameroon restricted the flow of COVID-19 information to the public. Various means of communication including telephone hotlines, electronic and print media, the internet, exhibitions, advertisements, etc., are used in creating awareness on the fight against COVID-19. The WHO African region makes a special educational COVID-19 video that is broadcasted on television; with special slots dedicated to the sensitization of a TV channel solely for informing about COVID-19. Due to electricity shortage, many people will not be opportune to watch TV and as such will depend on radios that are rechargeable. The ministries of health of most African countries set up telephone hotlines for public enquiry. In addition, partners and local authorities hold health roving exhibitions at health centres, railway stations, arranged health talks at communities and conduct mass public health education campaigns using posters, pamphlets, exhibition boards, etc., to inform the public [1].

With the rise of COVD-19 pandemic in Africa, a huge propaganda machinery is essential to be activated to inform the public. Ways used to motivate and inform the community in Africa to protect themselves and fight against COVID-19 includes but not limited to health educational programmes, songs, banners, COVID-19 advertisements on buses and daily press conferences by the governments [1]. With the Pandemic having started in China and spread to Europe and America, it shows that while the measures applied in most African countries are generally similar to those applied in the other countries of Europe and America, yet they were initiated at a later stage. As COVID-19 has spread, so has misinformation - fueling discrimination and stigma [4]. “Today, we have an open platform that allows amateur scientist, journalists and bloggers to express themselves through agenda setting, innuendo, half-truths, rumor and hearsay,” [4]. Just as the implications of COVID-19 are the new normal, so is the disinformation of reporting it. Distortion has been shown to run rife and is difficult to check it. For these reason social media platforms provide access and opportunity to spread misinformation and rumors easily. Misinformation about COVID-19 and harmful content have been major challenges for social media platforms in this era of COVID-19 Pandemic. Also, trusting content is especially critical as more people are using social media for news and updates, thus, there is a pertinent need for digital social media platforms such as Instagram, Facebook, Twitter and YouTube need momentous considerations to address misinformation about COVID-19. African Government officials and public leaders who are dealing with this global health crisis daily basis are equally responsible for providing accurate, scientific information on a regular basis in combating misinformation and rumor. This is because, if the accurate scientific information is not provided by public officials as seen in some African countries, crisis leaders on a regular basis during press releases, this provides an opportunity for the spread of misinformation.

**T: Treatment of COVID-19:** there is no specific antiviral treatment recommended for COVID-19, and no vaccine is currently available [1]. The treatment is symptomatic, and oxygen therapy represents the major treatment intervention for patients with severe infection. Mechanical ventilation may be necessary in cases of respiratory failure refractory to oxygen therapy, whereas hemodynamic support is essential for managing septic shock. In the desperate search to find effective treatments for COVID-19, two antimalarials hydroxychloroquine (HCQ) and chloroquine (CQ) have demonstrated antiviral activity against severe acute respiratory syndrome-coronavirus 2 (SARS-CoV-2) in vitro and small, poorly controlled or uncontrolled clinical studies. Hydroxychloroquine and azithromycin are effective in the treatment of COVID-19 [5]. The issue is fake drugs start circulating in African markets as a result of socio-economic factors. Also, the rush for chloroquine began after French researchers announced that half of 14 coronavirus patients who underwent therapy with the anti-malaria drug got better [5]. Cameroon was notified on March 9 by the WHO of the circulation of counterfeit anti-malaria drugs in its territory and neighboring Chad and Nigeria [6]. This aspect has heightened debates in many African countries on how to find local treatments, not just from African scientists but to also start to look into the many traditional herbal medicines and plants which have been used for centuries across different cultures on the continent. The COVID-19 pandemic in Africa has also triggered a rush for herbal formulas, onions, lemons, and ginger in the belief that they can protect against the virus [7]. The highest profile example so far has been Madagascar with its herbal tonic “COVID-Organics”, which is being championed strongly by island state’s president Andry Rajoelina [7]. The remedy, which contains the Artemisia annua (Sweet wormwood) plant often used to treat malaria is being ordered by other African countries. In Cameroon, a Catholic Church archbishop says he has discovered a remedy for the coronavirus based on his 30 years of research into herbal medicine [8]. Archbishop Samuel Kleda, a naturotherapist claims have attracted plenty of attention in Africa as he’s been offering the “Essential Oils” remedy for free [8].

**H: Hospital containment measures:** African countries isolate probable and suspect COVID-19 cases, either in a designated health facilities, as is the case in Cameroon or in separate triage facilities, for example, at hospitals specific wards to screen and separate symptomatic patients at the first point of presentation [1]. African countries developed detailed infection control guidelines both for the hospital setup and for health care workers (HCWs) and conducted special infection control WHO training courses both online and offline for their HCWs [1]. Some African countries have hospital infection control teams, which monitor the infection control practice in hospitals and the proper use of personal protective equipment (PPE) by HCWs [1]. Many African countries faced acute shortage of medical masks and other protective equipment. Reinstituting infection control, contact tracing and active surveillance in hospitals can contribute significantly in controlling the COVID-19 outbreak [2]. Additional measures to prevent the transmission of COVID-19 outside hospitals includes the restriction of HCWs to work at one health care institution only and stay under work/home quarantine as well as restricting the visit of COVID-19 patients in hospitals [1].

**Q: Quarantine:** a study by Cascella et al. [9], suggests that the use of isolation is the best way to contain the COVID-19 Pandemic for individual countries. Being quarantined in Africa is quite kaleidoscopic. Mandatory quarantine of close contacts is a good measure for African countries experiencing local COVID-19 transmission, only the extent may differ. WHO established, a 14-day home quarantine of close contacts of probable and suspect COVID-19 cases [1]. In addition to home quarantine, people in are also quarantined in groups, e.g. in appropriate hospitals, government low-cost housing, hotels, stadiums, etc. They have been reports of people escaping quarantined centres in some African countries [9]. In African cultures, the sick are treated and revered, not isolated, making self-quarantining a difficult trigger to pull. Also, psychological trauma is a challenge, just being with ones thoughts alone for 14 days can be ironically bad, good, and, ugly the same time. However, the quarantine period can be a time to self-develop as much as possible (through meditation and reading) which is a healthy form of distraction. It is in this light that, the services of psychologist should be highly solicited in such moments, ensuring a psychologist call in to check on the quarantined persons at least twice daily (morning and night), thus giving positive reassurance.

**C: Community containment measures against COVID-19:** community containment measures aimed at limiting social interaction and movement of people have been implemented in most African countries. Most African countries closed schools and students placed under home quarantine. While “lockdown” isn’t a technical term used by public-health officials, it could refer to anything from mandatory geographic quarantines to non-mandatory recommendations to stay at home, closures of certain types of businesses, or bans on events and gatherings. In Africa, it is difficult as earlier mentioned due to poverty as many people have to sustain their daily livelihood by going out. Public places such as bars, public libraries, film cinemas, indoor sports complexes, etc. By early April 2020, African countries made a strong move, to mobilise its population in a fight against COVID-19 and developed a people’s surveillance system where family members, neighbours, etc., monitored each other to ensure that COVID-19 cases were identified quickly [10]. Additional measures to enhance early detection of cases in the community and to reassure the public included public temperature screening, for example, before entering some major cities, schools, public buildings, offices, hospitals, etc. In Cameroon, people are asked to wear masks in closed public places. There is need to minimise economic impact and social disruption through multisector, international, community- collaborative, bottom-top and narrative inquiry approaches from a socio-ecological model. In Africa, socialisation is not just a matter of culture but survival. “People need to work, ” which involves taking public transport and mingling with other passengers in oft-packed buses. This equally puts community containment measures against COVID-19 in jeopardy.

**C: Case detection and contact tracing of COVID-19:** a big setback in Sub-Saharan African countries preparing for a COVID-19 outbreak is the cost required to prepare health systems to detect and respond to the disease [1]. Early case detection followed by rapid and effective isolation is a key measure to control COVID-19 spread [2]. All African countries affected by local COVID-19 transmission instituted diverse intensive case finding activities, which began with alerting health care providers and providing a case definition and diagnostic protocols. Contract tracing is a core public health function that public health agencies have done for years [1]. Contact tracing helps to limit COVID-19 transmission when the first cases are identified within a country but can be very resource intensive [10]. It is likely not to be feasible when community transmission is occurring and cases outside known transmission chains increase greatly. Africa Centres for Disease Control and Prevention recommended African member States to use the characteristics of the epidemic in their country to decide when and how to do contact tracing [10]. As such all African countries affected by COVID-19 started epidemiologic investigation of probable and suspect cases as soon as possible, by interviewing cases for exposure, travel and contact history, followed by active contact tracing of close contacts. During contact tracing, the contacts of the infected person are generally called up, asked if they’re feeling sick, and advised to self-quarantine for a period of time.

The control measures implemented by countries worldwide closely resemble each other and are generally in line with the WHO recommendations. However, differences among the countries can be seen in the timeliness of implementation and in the mode and extent to which individual countries apply or enforce control measures. This is seen in African countries generally with weak health systems. Despite all these concerns, Africa ought to have some advantages over the COVID-19 Pandemic that has scorched countries in Asia, Europe, and the Americas. On the other hand, the fact that COVID-19 spread to African countries later than other regions around the world had to be considered an advantage in combating the COVID-19 pandemic. The rate of spread, and level of impact of the COVID-19 Pandemic had to be recognized early, and measures such as suspension of international flights, limited curfew practices, suspension of schools, , and quarantine implemented relatively at an early stage. In line with Africa CDC, we reiterate that the most successful response to COVID-19 in Africa must consider context and adaptability, and must be data-driven. We recommend the strengthening public health systems for immediate response and for a lasting recovery; Monitor data on how public health and social measures meet local COVID-19 conditions and needs. Non-SHEF2 model related control and containment measures which include seven key measures against COVID-19 in Africa doped ‘TITHQC2’ namely, T: Travel-related measures, I: Information and guidance, T: Treatment; H: Hospital containment measures; Q: Quarantine, C: Community containment measures, C: Case detection and contact tracing has global health impact. A dilemma of trying to maintain socio-economic stability while at the same time protecting public health would challenge any African nation. The COVID-19 Pandemic has enhanced indications of how dire the consequences of a highly infectious disease Pandemic could be. The significance of timely preparedness and establishing sensitive active and passive surveillance systems information is perhaps the main lesson that the COVID-19 is teaching us. In line with the steps being taken across the globe to control and contain COVID-19 Pandemic, African countries should strictly adhere to the Non-SHEF2 model.

## Competing interests

The authors declare no competing interests.
